# Safe and promising outcomes of in-hospital preoperative rehabilitation for coronary artery bypass grafting after an acute coronary syndrome

**DOI:** 10.1186/s12872-024-03757-7

**Published:** 2024-03-04

**Authors:** Ken Nakamura, Shusuke Arai, Kimihiro Kobayashi, Shingo Nakai, Ri Sho, Ai Ishizawa, Daisuke Watanabe, Shuto Hirooka, Eiichi Ohba, Masahiro Mizumoto, Yoshinori Kuroda, Cholsu Kim, Hideaki Uchino, Takao Shimanuki, Tetsuro Uchida

**Affiliations:** 1 Division of Cardiovascular Surgery, Nihonkai General Hospital, Sakata, Japan; 2 Second Department of Surgery, Faculty of Medicine, Yamagata University, Yamagata, Japan; 3 Department of Public Health, Faculty of Medicine, Yamagata University, Yamagata, Japan

**Keywords:** Coronary artery bypass grafting, Cardiac rehabilitation, Acute coronary syndrome, Acute heart failure, Acute myocardial infarction

## Abstract

**Objective:**

In patients with stable hemodynamic status after an acute coronary syndrome (ACS), coronary artery bypass grafting (CABG) after preoperative investigations can provide outcomes comparable to those of emergency surgery. However, no established guidelines exist regarding the preparation period before surgery. We report the results of the use of an inpatient cardiac rehabilitation program followed by CABG after an ACS to improve post-operative outcomes and prognosis after discharge.

**Methods:**

From 2005 to 2017, 471 patients underwent either isolated or combined CABG at our institution, and of those, the 393 who received isolated CABG were included in the analysis. Twenty-seven patients (6.9%) were admitted with ACS and underwent preoperative rehabilitation before undergoing CABG, with a subsequent review of surgical morbidity and mortality rates. Propensity score matching yielded a cohort of 26 patients who underwent preoperative rehabilitation (group A) and 26 controls (group B). Preoperative characteristics were similar between groups.

**Results:**

The completion rate of the rehabilitation program was 96.3%. All programs were conducted with inpatients, with an average length of stay of 23 ± 12 days. All patients completed in-bed exercises, and 85% completed out-of-bed exercises. The 30-day postoperative mortality was 0% in both groups A and B, and the rate of postoperative major adverse cardiac or cerebrovascular events at 12 months did not differ significantly between groups (7.7% vs 3.9%, respectively; *p* = 1.0). The duration of mechanical ventilation (1.3 ± 0.3 vs 1.5 ± 0.3 days, respectively; *p* = 0.633), length of intensive care unit stay (4.4 ± 2.1 vs 4.8 ± 2.3 days, respectively; *p* = 0.584) and length of hospital stay (25 ± 13 vs 22 ± 9 days, respectively; *p* = 0.378) did not differ significantly between groups.

**Conclusions:**

No complications of preoperative rehabilitation were observed, suggesting that it is an acceptable option for patients who experience ACS and undergo CABG. These results are promising in offering more robust designs of future trials.

## Introduction

In the treatment of patients with ST elevation myocardial infarction (STEMI), the most important factor is prompt revascularization, and today, based on advances in percutaneous coronary intervention (PCI) techniques, emergency coronary artery bypass grafting (CABG) is rarely required [1, 2]. Even if acute coronary syndrome (ACS) requires early CABG, if the hemodynamic status is stable, it is preferable to perform CABG several days after the initial treatment, with sufficient preoperative examination [3], and the treatment outcome is not different from that of early CABG [4]. There is no standard method for the timing of surgery or preoperative conditioning of patients who cannot be discharged from the hospital after ACS or associated acute heart failure but can be scheduled for CABG on a standby basis, and the choice is individualized according to the patient's condition.

Rehabilitation after cardiac surgery is reported to reduce total mortality significantly [5], especially after CABG, and to reduce the incidence of rehospitalization and cardiovascular events [6]. Studies on preoperative rehabilitation have been reported in the past, and there is a consensus that it is effective for elderly patients at relatively low risk [7–9]. However, the expansion of its application to high-risk patients and younger patients remains a challenge, and its position as a standard treatment method has not yet been established. We actively provide preoperative rehabilitation to patients scheduled and waiting for CABG after an event due to ACS to wean them off bedrest early after surgery, discharge them from the hospital, and maintain their activity. The purpose of this study was to evaluate whether a physiotherapy treatment regimen initiated in the preoperative phase could prevent postoperative respiratory and musculoskeletal complications and influence the prognosis after cardiac surgery. In this report, we describe our treatment results.

## Patients and methods

### Study design

This is a single-center, retrospective observational study. The study’s retrospective was approved by the ethics committee of Nihonkai General Hospital, and conformed to the Declaration of Helsinki. The requirement for informed consent was waived by Ethics committee of Nihonkai General Hospital based on the study’s retrospective analysis of patient data.

### Setting and participants

**Fig. 1:**
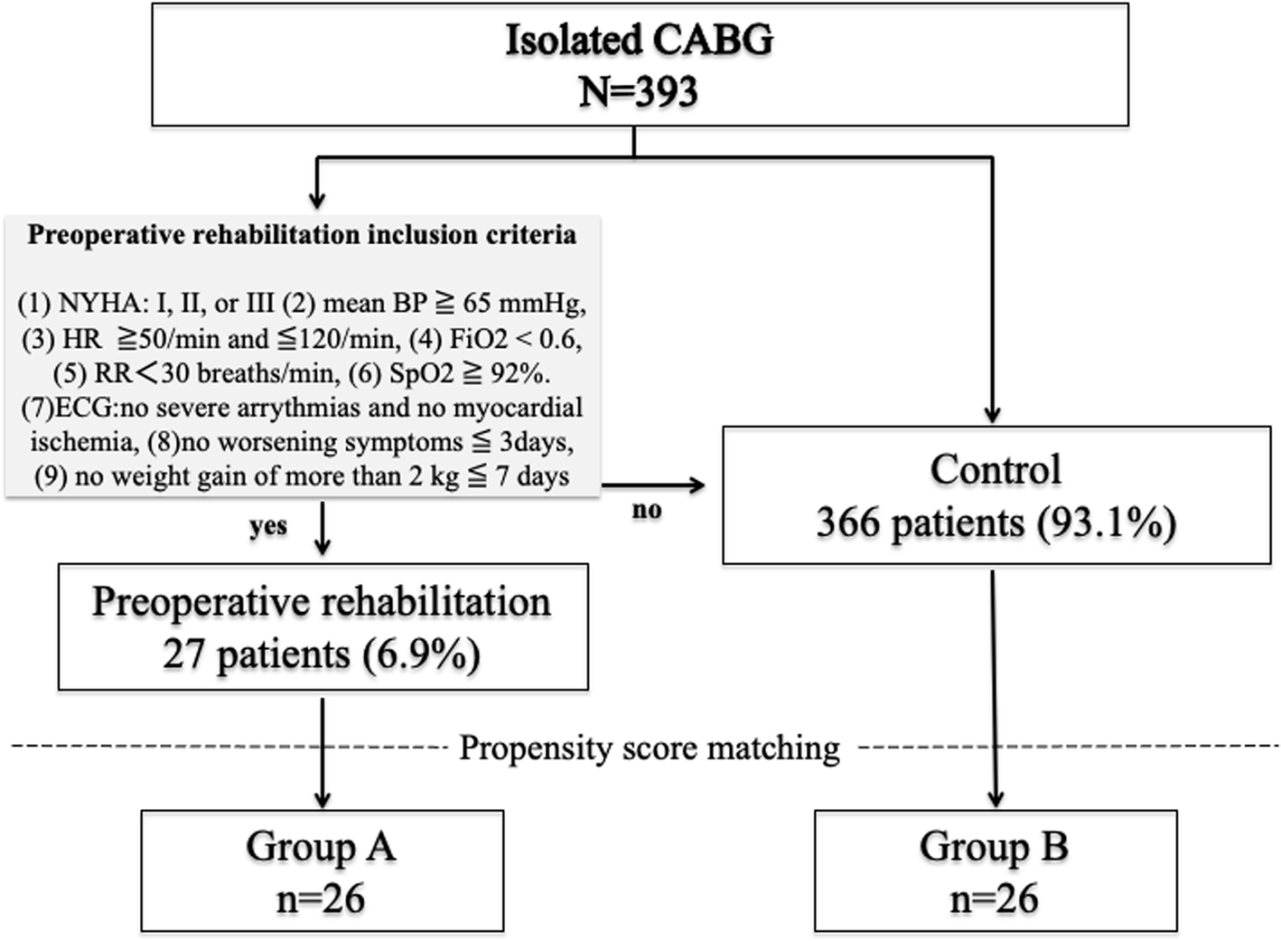
Summary flow diagram of patient disposition

This study conducted at Nihonkai General Hospital, and involved 471 unique patients from December 2005 to December 2017 who underwent isolated or combined CABG. Of those, 393 patients who underwent isolated CABG were included in the analysis. Prior to all CABGs, a multidisciplinary team of cardiothoracic surgeons, cardiologists, physical therapists, nurses, and medical technicians met to discuss the indication for preoperative rehabilitation and preoperative conditioning. Inclusion criteria adopted to enroll the patients in this study were ACS, a New York Heart Association (NYHA) class I, II or III, good exercise tolerance before in-hospital admission, and chance to benefit from respiratory and physical excercise. The inclusion criteria for patients indicated for the preoperative rehabilitation program were similar to the guidelines developed by the Japanese Society of Cardiology [10]: a mean blood pressure greater than 65 mmHg, a sustained pulse greater than 50 and less than 120, and the absence of the appearance of new severe arrhythmias and ECG changes suggestive of myocardial ischemia. Patients were eligible for treatment if their respiratory rate was less than 30 breaths/min, SpO_2_ was greater than 92%, and FiO_2_ was less than 0.6. The following conditions were also considered for eligibility for the rehabilitation program: no worsening of subjective symptoms within the past 3 days, no weight gain of more than 2 kg within the past week, and no NYHA functional class IV heart failure (Fig. 1). The evaluation of exercise tolerance prior to the start of the program began with an interview to ascertain psychological status, cognitive ability, exercise capacity, and NYHA cardiac function classification. If exercise testing is possible, cardiopulmonary exercise (CPX) testing should be performed and evaluated. If it is difficult to perform exercise testing, a rating of perceived exertion (RPE) should be evaluated based on the Borg index [11] and talk test, using a heart rate of less than 120 bpm as a guide. A Borg index of 11 to 13 was used as a guide to determine RPE.

The cardiac rehabilitation program is a comprehensive program that includes nutritional guidance, psychological counseling, and management of coronary risk factors (blood pressure, lipids, diabetes, smoking, etc.) in addition to exercise therapy. Rehabilitation is based on an acute weaning program, gradually increasing in intensity from head-up in bed, end-sitting, and standing at the bedside, with the goal of walking to the toilet and walking freely in the ward [12]. Patients who complete the weaning program without progression of their condition are prepared for cardiac surgery with low-intensity aerobic exercise and resistance training.

### Variables

The primary outcome was the occurrence of postoperative major adverse cardiac and cerebrovascular events (MACCE), which included death, acute myocardial infarction, cerebrovascular event, or further revascularization by percutaneous coronary intervention or CABG. Intraoperative findings, such as the amount of inotropic agent used during surgery, the presence or absence of mechanical circulatory support, and difficulty in weaning from cardio-pulmonary bypass, were evaluated as secondary outcomes, as were the number of days until extubation, postoperative complications, and postoperative hospital stay. Postoperative atrial fibrillation, mediastinitis, intubation time longer than 72 h, intensive care unit (ICU) stay longer than 7 days, and postoperative length of stay longer than 30 days were also included.

### Data sources/measurements

The occurrence of any short runs of atrial fibrillation more than 30 s during the hospital stay was considered to represent an occurrence of atrial fibrillation. A neurologic event was defined as an endpoint when symptoms appeared and could be confirmed using computed tomography (CT) and magnetic resonance imaging (MRI). The final diagnosis was performed by a neurosurgeon, and it was considered a neurologic event if diagnosed. If there were no visual findings, a transient ischemic attack was not included. These definitions are similar to those presented previously [13].

As previously reported, our institution performs prophylactic intra-aortic balloon pump (IABP) therapy in all high-risk patients [13]. An IABP was inserted in the catheterization lab the day prior to CABG, with continuous intraprocedural IABP therapy and use postoperatively as clinically indicated; patients were monitored in the ICU pre- and post-operatively.

In addition, we treated dental conditions, glycemic imbalances, and co-morbid treatable diseases, such as carotid artery stenosis, prior to cardiac surgery. A cardiopulmonary bypass (CPB) circuit was used when considered necessary at a preoperative conference. Its use was suggested mainly by preoperative characteristics (e.g., a large left ventricle, low cardiac function), and on-pump CABG was scheduled based on comprehensive risk assessment, such as the location or quality of the target vessels and technical challenges. If it was judged that a complete revascularization was feasible on the beating heart, off-pump CABG (OPCAB) was scheduled. Conversion to CPB was considered if there was any evidence of hemodynamic instability concerns, such as ventricular arrhythmia, hypotension (systolic pressure ≤ 80 mmHg), and cardiac arrest during the OPCAB procedure. OPCAB was performed after a median sternotomy. The heart was displaced using a posterior pericardial stitch, gauze and a tissue stabilizer (Octopus; Medtronic Corporation, Minneapolis, Minn., USA), along with body position changes and gravity support (Trendelenburg, right and left table rotations). A CO_2_ blower/NaCl misting device was used in situations where a bloodless field was not achieved after proximal target vessel occlusion. On-pump isolated CABG was performed with almost the same technique. All on-pump CABG was carried out in the beating condition. Grafting was always performed from the left internal mammary artery to the left anterior descending coronary artery, followed by grafting of the circumflex coronary artery and the right coronary artery using a radial artery or a saphenous vein. The bilateral internal mammary arteries were used in the non-touch aorta technique if ascending aortic sclerosis or calcification was assessed based on preoperative findings from imaging examinations and intra-operative palpation. The quality of the anastomosis was assessed after grafting with the use of a transit-time flow probe (Butterfly Flowmeter; Medistim, Oslo, Norway).

### Bias

These items were confirmed with bivariate analysis to avoid selection bias.

## Statistical analysis

Continuous variables were expressed as the mean and standard deviation or the median and interquartile range, 95% confidence interval, and categorical variables were expressed as frequencies or percentages. Matched-group analysis was performed by propensity matching between patients with preoperative rehabilitation and controls. Propensity scores were generated in two steps using logistic regression analysis. Potential predictors were selected from published data review, known confounding covariates for the outcome of interest, differences between the two patient groups (Table 1), and clinical judgment. Continuous data were analyzed with analysis of variance (ANOVA), an independent Student’s t-test or the Mann–Whitney U-test. Categorical variables were analyzed with a chi-square analysis and Fischer’s exact test. The MACCE-free rates after surgery for the two groups were determined by Kaplan–Meier survival analysis and compared with the log-rank test. Analyses were conducted with JMP software, version 17.0 (SAS Institute Japan, Tokyo, Japan).

**Table 1:**
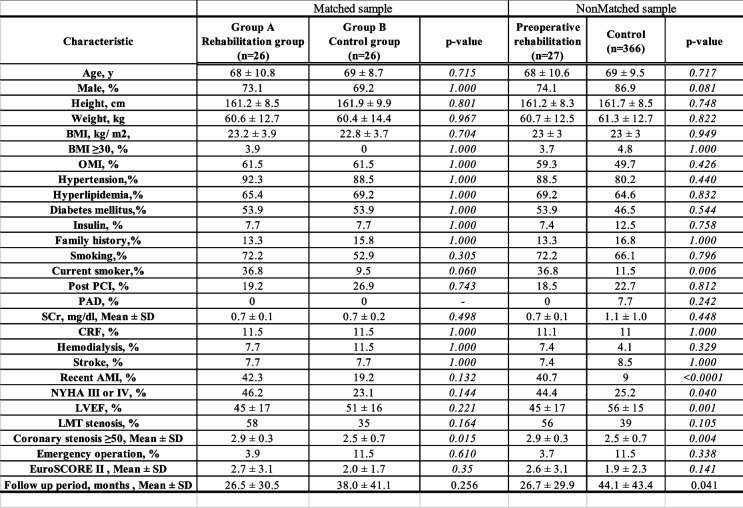
Baseline patient characteristics (preoperative data)

*SD* Standard deviation, *IQR* Interquartile range, *BMI* Body mass index, *OMI* Old myocardial infarction, *PCI* Percutaneous coronary intervention, *PAD* Peripheral arterial disease, *SCr* Serum creatinine, *CRF* Chronic renal failure, *AMI* Acute myocardial infarction, *NYHA* New York Heart Association, *LVEF* Left ventricular ejection fraction, *LMT* Left main coronary trunk, *EuroSCORE* European system for cardiac operative risk evaluation

## Results

A total of 393 consecutive patients were included in this study (27 who received preoperative rehabilitation and 366 controls). Patient preoperative clinical data are listed in Table 1. Before matching, when the preoperative rehabilitation group was compared with the control group, the preoperative rehabilitation group had more current smokers (36.8% vs. 11.5%, respectively; *p* = 0.006), more patients with an NYHA classification of III or IV (44.4% vs. 25.2%, respectively; *p* = 0.040), lower EF (45% ± 17% vs. 56% ± 15%, respectively; *p* = 0.001), and more severe coronary stenosis (2.9 ± 0.3 vs. 2.5 ± 0.7, respectively; *p* = 0.015). After matching, there was no difference between groups in baseline characteristics. The Euro SCORE II was 2.7 ± 3.1 vs. 2.0 ± 1.7, respectively (*p* = 0.35), and the left ventricular ejection fraction (LVEF) was 45% ± 17% vs. 51% ± 16%, respectively (*p* = 0.221). The mean follow-up time was 26.5 ± 30.5 months vs. 38.0 ± 41.1 months, respectively. Twenty-seven patients met the inclusion criteria and were indicated for preoperative rehabilitation after ACS, of whom 40.7% had a diagnosis of acute myocardial infarction (AMI; 40.7 vs. 9.0%, respectively; *p* < 0.0001) when hospitalized. Only one patient (3.7%) had a complication of stroke immediately before the start of rehabilitation, and acute heart failure was present in all patients. The completion rate of the rehabilitation program was 96.3%. In only one case, left bundle branch block appeared on an electrocardiogram 5 days after the start of the program, and emergency CABG was performed. All rehabilitation programs were conducted with inpatients, with an average length of stay of 23 ± 12 days. All patients completed in-bed exercises, and 85% completed out-of-bed exercises. Eighty-five percent of the patients were able to leave the ICU and move to a ward (Table 2).

**Table 2:**
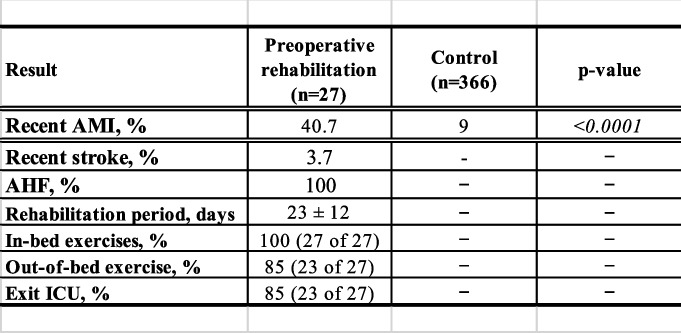
Indications for and results of preoperative rehabilitation

*AMI* Acute myocardial infarction, *AHF* Acute heart failure, *SD* Standard deviation, *IQR* Interquartile range, *ICU* Intensive care unit, *in-bed exercises* Completion of in-bed exercises, *out-of-bed exercise* Completion of out-of-bed exercise

**Fig. 2:**
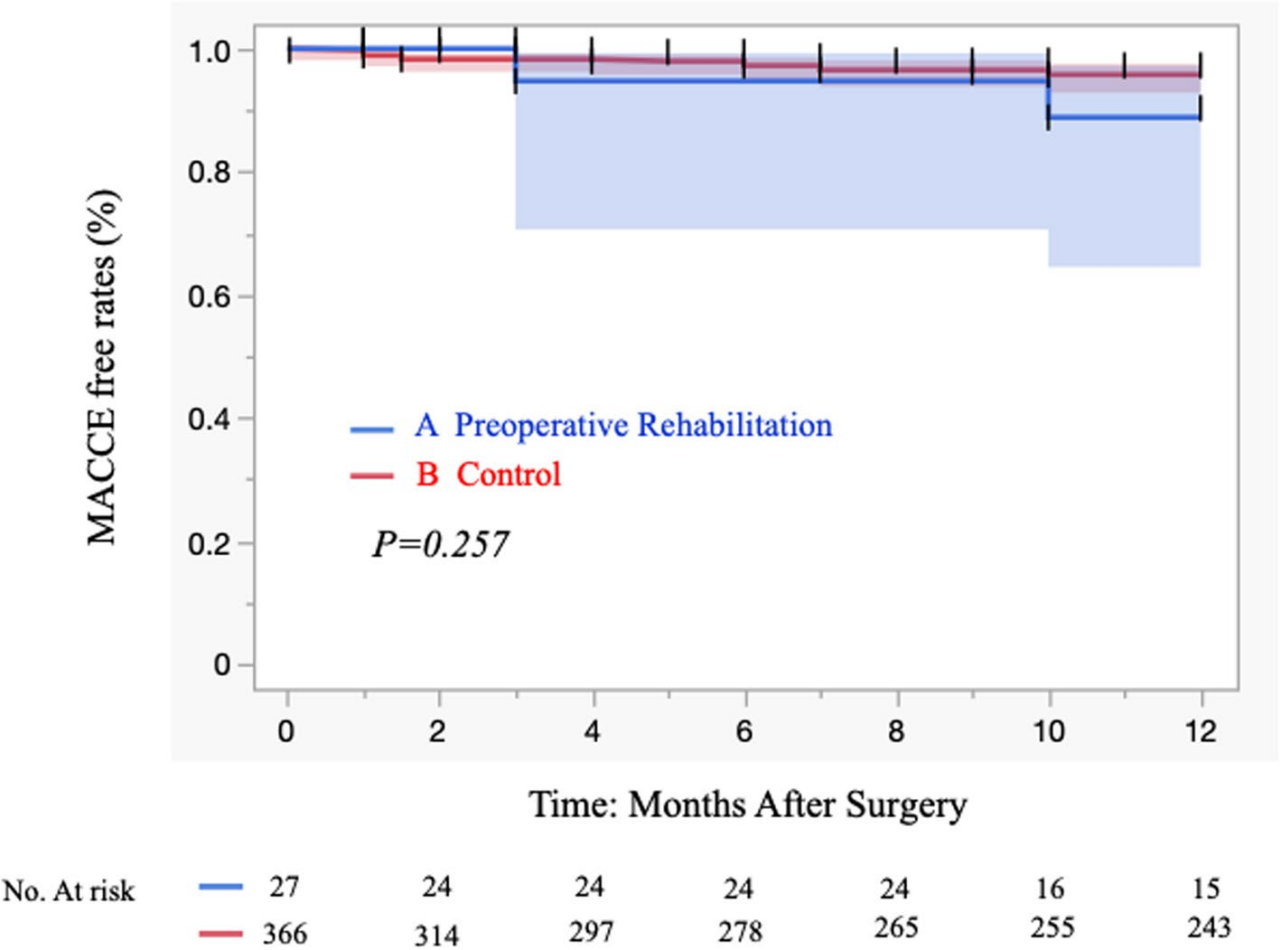
Kaplan–Meier curves of MACCE-free rates of 393 patients with isolated CABG in our institution: 27 patients who received preoperative rehabilitation and 366 control patients

**Fig. 3:**
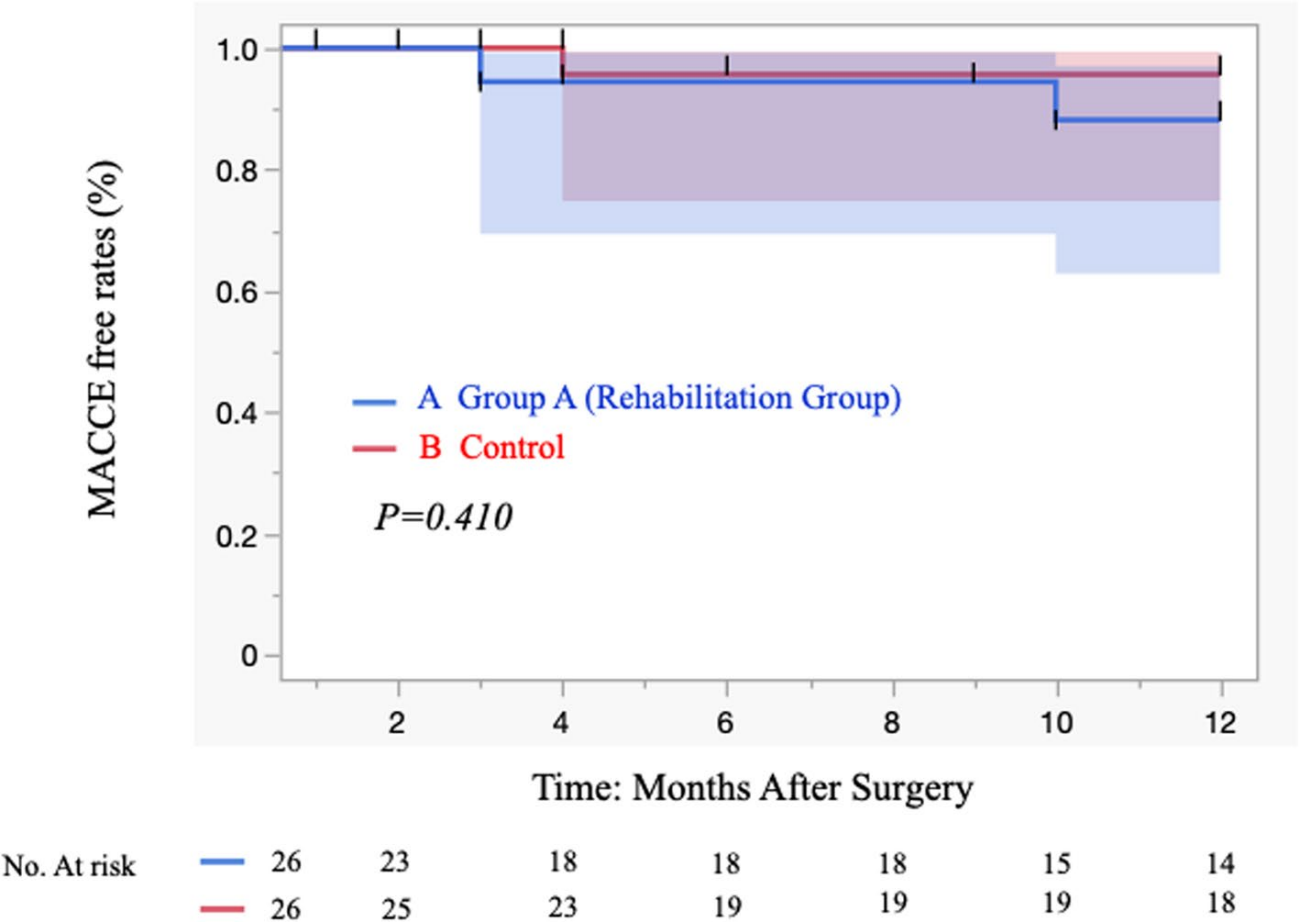
Kaplan–Meier curves of MACCE-free rates of 52 propensity score-matched patients with isolated CABG in our institution: 26 patients who received preoperative rehabilitation and 26 control patients

At the induction of anesthesia and just before the start of surgery, preoperative rehabilitation group compared with control group tended to have a lower cardiac index (2.5 ± 0.8 vs. 2.8 ± 0.7, respectively; *p* = 0.041) (Table 3). Intra- and postoperative results are shown in Table 4. There were no significant differences between groups in operation time, use of cardiopulmonary bypass, pump time, conversion to on-pump CABG, reoperation for bleeding, required transfusion of red blood cells, leg wound problems, leg wound infection, occurrence of mediastinitis and neurologic events. When comparing groups A and B, the duration of mechanical ventilation (1.3 ± 0.3 days vs 1.5 ± 0.3 days, respectively; *p* = 0.633), the length of ICU stay (4.4 ± 2.1 days vs 4.8 ± 2.3 days, respectively; *p* = 0.584) and the length of hospital stay (25 ± 13 days vs 22 ± 9 days, respectively; *p* = 0.378) were not significantly different. Inpatient surgical mortality, 30-day MACCE and 30-day mortality were 0% in both groups. The postoperative MACCE-free rate (1 year) was 92.3% (group A) vs 96.1% (group B) (*p* = 1.0) and 92.6% (rehabilitation group) vs 96.4% (control group) (Figs. 2, and 3). Specifically, two patients in group A died after discharge; one died of exacerbation of interstitial pneumonia, and the other died while being treated for a urologic malignancy.

**Table 3:**
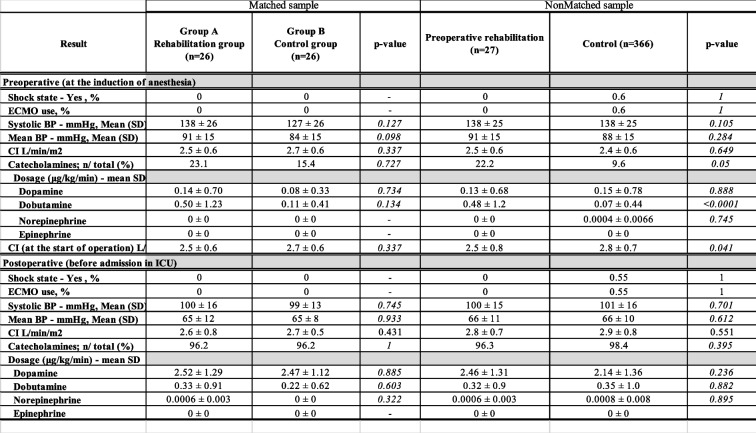
Clinical outcomes: pre- and postoperative data with or without preoperative rehabilitation

*ECMO* Extracorporeal membrane oxygenation, *BP* Blood pressure, *SD* Standard deviation, *CI* Cardiac index, *ICU* Intensive care unit

**Table 4:**
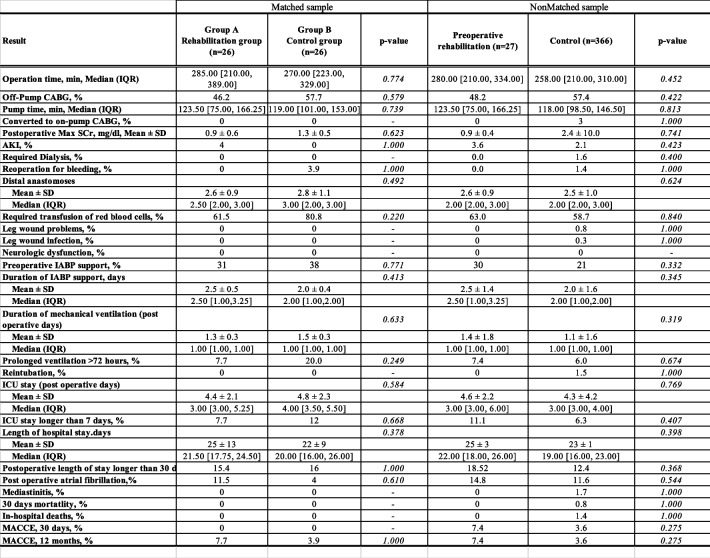
Clinical outcomes and complications with or without preoperative rehabilitation

*IQR* Interquartile range, *SD* Standard deviation, *CABG* Coronary artery bypass grafting, *SCr* Serum creatinine, *ICU* Intensive care unit, *MACCE* Major adverse cardiac and cerebrovascular events

## Comment

Exercise therapy after cardiac surgery has proven to be effective by various indices, including exercise tolerance, cardiac and peripheral function, and mental health [14, 15]. Only rehabilitation after CABG is said to improve long-term outcomes after cardiac surgery, with significant improvements in MACCE, total number of rehospitalizations, and number of rehospitalizations due to cardiac disease [6]. In this study, we found that indicators of cardiac function, such as blood pressure and cardiac index, tended to be lower in the preoperative rehabilitation group at induction of anesthesia, but i) there was no difference in the number of postoperative days of mechanical ventilation, ICU stay, and hospital stay, and ii) results for early postoperative mortality and MACCE occurrence within 1 year were favorable. Although these results do not directly indicate the effectiveness of rehabilitation prior to cardiac surgery, they are favorable, because they show that the postoperative outcomes of patients with complications of ACS immediately prior to surgery are non-inferior to those of patients who underwent conventional cardiac surgery.

Rehabilitation programs for acute heart failure are well established [12], and rehabilitation for elderly patients with heart failure [16], in particular, has been shown to be effective in reducing hospital stay, maintaining activities of daily living (ADL) at discharge, and reducing rehospitalization [17]. What became clear in our study was the novelty of continuing rehabilitation and surgery in patients in the hospitalized state after ACS, which can be safely completed, and that even high-risk patients with low cardiac function and acute heart failure can be treated, with outcomes that are not significantly different from those of patients who undergo scheduled surgery.

The goal of our rehabilitation program was to prevent postoperative decline in physical function and deconditioning due to prolonged periods of bed rest prior to surgery [18]. Our first step in achieving this goal is to educate patients so that they can manage their own lives based on motivation. Thorough patient education on this step has been reported to be effective in reducing rehospitalization for heart failure and improving life expectancy [19, 20]. This involves patient education across multiple professions, including not only physicians and physical therapists, but also pharmacists and nutritionists.

CPX testing is used at our facility to assess exercise tolerance, but if the patient's condition does not allow it, heart rate during exercise, a talk test, and the RPE are used as evaluation indices. When the RPE is used as the evaluation index, the Borg index is used, with an upper limit of 11–13. These are recommended in the guidelines of the Japanese Society of Cardiology [10]. Exercise stress tests are not performed if the patient is NYHA class IV or unstable, or if exercise stress would cause a rapid deterioration of the patient's general condition. However, the decision is made solely on an individual case-by-case basis when the benefits of exercise loading are judged to outweigh the risks. Exercise therapy consisted mainly of aerobic exercise, resistance training, and stretching [21], which we also performed. Resistance training focused on isotonic contraction rather than isometric contraction, which is also recommended for rehabilitation after cardiac surgery [22].

The purpose of our study was to evaluate the safe performance of physiotherapy treatment regimen initiated in the preoperative phase in patients who had an ACS event prior to cardiac surgery could prevent postoperative respiratory and musculoskeletal complications and influence the prognosis after cardiac surgery. Therefore, the findings evaluated as outcomes in acute heart failure rehabilitation were set as endpoints for the present study. The objectives of cardiac rehabilitation for acute heart failure are (1) to control the adverse effects of excessive bed rest, such as physical deterioration, delirium, and cognitive decline, by early release from bed and (2) to develop and implement a plan for early and safe discharge. The MACCE, which evaluates long-term postoperative outcomes in coronary disease, was used for this evaluation, especially since the long-term prognosis has been shown to improve with continued exercise therapy after discharge from the hospital [23, 24]. Compared to average values, we observed better outcomes with regard to 30-day mortality (group A vs group B = 0 [0%] vs 0 [0%], respectively) and in-hospital deaths (0 [0%] vs 0 [0%], respectively). After 1 year, MACCE showed an increase in total mortality (group A vs. group B = 2 [7.7%] vs. 1 [3.9%], respectively); however, most deaths were not due to cardiac disease but to other diseases, such as pneumonia and malignancy.

### Limitations

This study has several limitations. First, the number of patients was relatively small. Significant differences in results may appear as the number of patients increases. Second, the study was performed at a single center; therefore, the results might not be generalizable to other centers in different situations. Third, the nonrandomized design might have affected our results, owing to unmeasured confounding factors, procedural bias, or detection bias. Fourth, since the method of assessment of exercise tolerance was chosen based on the patient's condition, the assessment was not uniform and may have affected the content of the program. These may be involved in observer bias. Sixth, there was no set goal for the extent to which rehabilitation should be achieved and surgery should be performed, and further study is needed.

In addition, a cost-effectiveness evaluation of hospitalization costs will be necessary in the future, as this program will increase the number of hospital days from the preoperative period. These are potential obstacles to the actual implementation of this study. While subsidized hospitalization costs in Japan make it easy to develop such a strategy, it is unclear whether similar programs can be replicated in other countries.

## Conclusion

Patients who underwent preoperative rehabilitation after the onset of ACS had a safe course without serious complications and subsequently underwent CABG. Patients who received preoperative rehabilitation showed no difference in prognosis or occurrence of complications compared with regularly treated patients. Based on the present results, a more robust design of this treatment strategy in the future may contribute to improved patient outcomes.

## Data Availability

The datasets used and/or analysed during the current study are available from the corresponding author on reasonable request.
